# Refractory testicular germ cell tumors are highly sensitive to the targeting of polycomb pathway demethylases KDM6A and KDM6B

**DOI:** 10.1186/s12964-024-01912-3

**Published:** 2024-10-31

**Authors:** Doha Shokry, Mehwish W. Khan, Christine Powell, Samantha Johnson, Brayden C. Rennels, Raya I. Boyd, Zhengyang Sun, Zeeshan Fazal, Sarah J. Freemantle, Maryanna H. Parker, Miranda D. Vieson, Jonathan P. Samuelson, Michael J. Spinella, Ratnakar Singh

**Affiliations:** 1 Department of Comparative Biosciences, University of Illinois Urbana-Champaign, 2001 South Lincoln Avenue, Urbana, IL 61802 USA; 2 Department of Anatomy and Embryology, Alexandria University, Alexandria, Egypt; 3 Department of Pathobiology, University of Illinois Urbana-Champaign, Urbana, IL 61801 USA; 4 Department of Veterinary Clinical Medicine, University of Illinois Urbana-Champaign, Urbana, IL 61802 USA; 5 Carl R. Woese Institute for Genomic Biology, University of Illinois Urbana-Champaign, Urbana, IL 61802 USA; 6 Cancer Center of Illinois, University of Illinois Urbana-Champaign, Urbana, IL 61802 USA

**Keywords:** Testicular cancer, Polycomb, GSK-J4, GSK-126, Preclinical, Transcriptomics, Cisplatin, Chemotherapy resistance, Epigenetics

## Abstract

Testicular germ cell tumors (TGCTs) can be treated with cisplatin-based therapy. However, a clinically significant number of cisplatin-resistant patients die from progressive disease as no effective alternatives exist. Curative cisplatin therapy results in acute and life-long toxicities in the young TGCT patient population providing a rationale to decrease cisplatin exposure. In contrast to genetic alterations, recent evidence suggests that epigenetics is a major driving factor for TGCT formation, progression, and response to chemotherapy. Hence, targeting epigenetic pathways with “epidrugs” is one potential relatively unexplored strategy to advance TGCT treatment beyond cisplatin. In this report, we demonstrate for the first time that targeting polycomb demethylases KDM6A and KDM6B with epidrug GSK-J4 can treat both cisplatin-sensitive and -resistant TGCTs. While GSK-J4 had minimal effects alone on TGCT tumor growth in vivo, it dramatically sensitized cisplatin-sensitive and -resistant TGCTs to cisplatin. We validated KDM6A/KDM6B as the target of GSK-J4 since KDM6A/KDM6B genetic depletion had a similar effect to GSK-J4 on cisplatin-mediated anti-tumor activity and transcriptome alterations. Pharmacologic and genetic targeting of KDM6A/KDM6B potentiated or primed the p53-dominant transcriptional response to cisplatin, with also evidence for basal activation of p53. Further, several chromatin modifier genes, including *BRD4*, lysine demethylases, chromodomain helicase DNA binding proteins, and lysine methyltransferases, were repressed with cisplatin only in KDM6A/KDM6B-targeted cells, implying that KDM6A/KDM6B inhibition sets the stage for extensive chromatin remodeling of TGCT cells upon cisplatin treatment. Our findings demonstrate that targeting polycomb demethylases is a new potent pharmacologic strategy for treating cisplatin resistant TGCTs that warrants clinical development.

**Supplementary Information:**

The online version contains supplementary material available at 10.1186/s12964-024-01912-3.

## Introduction

Testicular germ cell tumors (TGCTs) are curable with traditional cisplatin-based chemotherapy [1]. However, cisplatin resistance occurs in approximately 15% of metastatic patients, with the majority dying from progressive disease [2]. No effective therapies exist for this patient population, and no clinically effective strategies exist to overcome cisplatin resistance, likely due to a lack of detailed understanding of the mechanisms responsible for cisplatin resistance in TGCTs [2, 3]. Further, testicular cancer is the most common carcinoma of males ages 15 to 45 and cisplatin-based cures are associated with many toxicities and co-morbidities, including ototoxicity, infertility, neuropathy, and a high risk of secondary cancers [4, 5]. Hence, cisplatin-sparing strategies for this young patient population are needed.

TGCTs are believed to arise from aberrant differentiation of primordial germ cells during development [6]. There is mounting evidence that TGCTs may be a cancer that is especially driven by epigenetic dysfunction, both in terms of etiology and response or resistance to chemotherapy [7, 8]. This includes a unique pattern of DNA hypomethylation and histone modifications compared to most other tumors due to their embryonic origins at a stage undergoing extensive epigenetic reprogramming, an association with *in utero* endocrine disruption and environmental stress, and a lack of driver DNA mutations [6, 9–11]. Due to their pluripotent primordial germ cell origins, TGCTs are known to have unique epigenetics compared with other solid tumors with unique patterns of DNA methylation, including prominent DNA hypomethylation and pluripotency-associated bivalent histone modifications [6, 7]. Particularly prominent is a lack of p53 mutations that is a proposed factor for high cisplatin curability. The possible unique reliance on distinct epigenetic drivers suggests that TGCTs may be uniquely sensitive to epigenetic-based therapies [7, 8].

Two major epigenetic pathways associated with target gene repression are CpG island methylation mediated by DNA methyltransferases DNMT1, DNMT3A, and DNMT3B and the polycomb pathway [12, 13]. There are two major polycomb repressive complexes (PRCs), PRC1 and PRC2. PRC2 contains the core components EZH2, SUZ12, and EED, while PRC1 is comprised of BMI1, CBX, RIN1A/B, and PCH [14]. EZH2 catalyzes H3K27 trimethylation, which is a docking site for the PRC1 complex that catalyzes monoubiquitination of H2A on K119 [14]. Both modifications repress gene expression. Additionally, the histone demethylases KDM6A and KDM6B remove H3K27 methylation [15]. The role of the PRC2 complex in cancer is complex, with the majority of studies suggesting that EZH2 promotes oncogenesis and is a pharmacologic cancer target [16]. However, there are several examples of documented tumor suppressor functions of PRC2 [17–19]. This complexity extends to whether polycomb represses or augments chemotherapy responses [20–23].

We and others have shown that TGCT cells are hypersensitive to low doses of hypomethylating agents (HMAs), such as decitabine and guadecitabine, including cisplatin-resistant cells, and that pretreatment with HMAs can reverse cisplatin resistance in cell and mouse xenograft models [24–28]. This strategy has been tested clinically with some promising patient responses in small trials [29, 30]. In addition, we generated a series of independent clonal, stable cisplatin resistant cell lines from three cisplatin hypersensitive parental human TGCT cell lines NT2/D1, 833K, and 2102EP [31–33]. The selection protocol was designed to mimic cisplatin therapy for TGCT patients namely as daily (day one to five) cisplatin administrations with repeated cycles every 21 days [1, 2]. Resistant clones were derived independently and demonstrated 3- to 30-fold cisplatin resistance in vitro and in vivo [32]. The cisplatin resistance in these TGCT isogenic cell models was associated with a coordinated decrease in EZH2, BMI1, and H3K27me3 levels coupled with a bi-directional shift between gene promoter and gene body DNA methylation among multiple gene sets resulting in an upregulation of polycomb target genes and a downregulation of tumor suppressor genes [31–33]. A gene signature based on polycomb target genes was also associated with recurrent and progressive disease in TGCT patients [32]. Further, DNMT3B levels were highly upregulated in cisplatin-resistant TGCT cells compared to isogenic parental cells, and DNMT3B-knockdown alone in parental cells was sufficient to induce H3K27me3, EZH2, and BMI1 levels and cisplatin hypersensitivity [33]. This suggests that DNA methylation and polycomb are coordinately regulated in TGCTs to modulate cisplatin sensitivity.

The apparent connectedness of DNA methylation and polycomb signaling alterations with cisplatin resistance, coupled with the demonstrated promising preclinical and clinical activity of DNA methylation targeting HMAs in TGCTs, prompted us to examine whether polycomb targeting may be a second epigenetic-based treatment for cisplatin-resistant TGCTs. We chose to target polycomb H3K27me3 demethylases KDM6A and KDM6B as these are the enzymes in mammalian cells that remove the H3K27me3 mark and in prior studies we have shown that lower levels of H3K27me3 is associated with cisplatin resistance in TGCT cells [14, 15, 32, 33]. Genetic and pharmacologic inhibition of KDM6A and KDM6B sensitized cisplatin-resistant and wild-type TGCT cells to cisplatin and produced dramatic synergistic tumor regression in animal models. This was associated with decreased expression of DNMT3B. Transcriptome analysis revealed a robust alteration in gene expression with polycomb demethylase targeting and basal and cisplatin-mediated potentiation of p53 target gene activation and downregulation of multiple chromatin-modifying enzymes in response to cisplatin. Our findings preclinically validate targeting polycomb demethylases KDM6A/B as a potent pharmacologic strategy for treating cisplatin-resistant TGCTs that warrants further preclinical and clinical investigation.

## Materials and methods

### Drug treatments and cell viability and proliferation assays

All cells were cultured in DMEM (Sigma) with 10% FBS (GeminiBio). The NT2/D1, 833K, and 2102EP cells are human testicular cancer-derived embryonal carcinoma cell lines and colon cancer HCT116, breast cancer MDA-MB-231 and MCF7, and glioblastoma U87-MG cell lines were all purchased from ATCC and authenticated by ATCC with karyotyping and short tandem repeat profiling, as described [24]. Cells were frozen within 1 month of purchase and used within 2 months of resuscitation. Derivation of cisplatin-resistant NT2/D1-A4 and 2102EP-C1 cells was previously described in detail [32].

Cells were treated with the indicated dosages of cisplatin (Sigma) for 6 h and cells assayed for survival 3 days later. For sequential treatments, cells were pretreated with the EZH2 inhibitor GSK-126 or the KDM6A/KDM6B histone demethylase inhibitor GSK-J4 (both from Selleck Chemicals) for 3 days at doses that alone did not affect viability by more than 5% (0.5 µM and 1.0 µM, respectively) and then treated with cisplatin [32]. To assess cell viability, CellTiter-Glo (Promega) assays were performed. For each cell line, three biological replicates were tested at each concentration, and experiments were repeated at least twice on different days.

### Lentiviral shRNA knockdown

Lentiviruses were produced by co-transfecting HEK293 cells with 10 µg of the viral packaging vector pCMV-dR8.2 and envelope vector pCMV-VSV-G (10:1 ratio) and 10 µg of lenti-shRNA or + targeting BMI1 (TRCN0000020155, TRCN0000020156, TRCN0000020157), EZH2 (TRCN0000018365, TRCN0000040074 and TRCN0000040075), KDM6A (TRCN0000107760, TRCN0000107761, TRCN0000107762, TRCN0000107763, TRCN0000107764) and KDM6B (TRCN0000359976, TRCN0000236678, TRCN0000236677, TRCN0000236676, TRCN0000236679), were purchased from Sigma along with pLKO.1-puro empty vector control (SHC001). The HEK293 cell medium was changed 24 h after transfection and cells were incubated for 48 h to allow for virus production. After 48 h, HEK293 medium containing viral particles was filtered and transferred onto 833 K, 2102EP, or NT2/D1 cells for 48 h. Cells were selected with 5 µg/ml puromycin. For dual KDM6A and KDM6B knockdown, a combination of two lentivirus pLKO.1-puro, pLKO.1-Neo (Addgene plasmid #13425), shRNA targeting KDM6A (TRCN0000107764-neo) and shRNA targeting KDM6B (TRCN0000236676) were used. 48-hour post-transduction cells were selected with 5 µg/ml puromycin and 250 µg/ml neomycin.

### Xenograft experiments

All animal experiments were approved by the University of Illinois Urbana-Champaign IACUC under protocol 24080. Under this protocol maximum allowable tumor size is 15 mm in any direction, which was not exceeded. For mouse studies, 5–8-week-old male athymic nude mice (Jackson Labs) were injected subcutaneously in the flank with 5 × 10^6^ 2102EP-C1 and NT2/D1 cells after resuspension in a 50:50 ratio of DMEM/Matrigel (Corning). Once palpable tumors were detected, tumor volume was measured twice weekly with calipers using the formula V= (L × W × W)/2. At a tumor volume of approximately 150 mm^3^ (day 1), mice were randomly assigned to vehicle (PBS), GSK-J4, cisplatin, or a combination of cisplatin + GSK-J4 treatments. GSK-J4 was given by intraperitoneal (IP) injection every other day for 6 total injections at 50 mg/kg (days 1, 3, 5, 7, 9 and 11). Cisplatin was given as a single IP injection on day 5 at 7.5 mg/kg for 2102EP-C1 cells and 6.0 mg/kg for NT2/D1 cells. In separate experiments, mice were injected with 5 × 10^6^ 2102EP-C1-pLKO.1 cells or 2102EP-C1-shKDM6A + shKDM6B (sh6A + 6B) cells. Once tumors reached a volume of 150 mm^3^ mice were randomly assigned to a single IP injection of either PBS or 7.5 mg/Kg cisplatin. Body weight was also measured twice weekly. Mice were sacrificed when tumors reached humane endpoints with euthanasia by carbon dioxide followed by cervical dislocation.

### RNA-sequencing

RNA was extracted from cisplatin-resistant NT2/D1-A4 and 2102EP-C1 cells pretreated with only 1.0 µM GSK-J4 for 3 days, treated with only 0.5 µM cisplatin for 6 h, or both. RNA was also extracted from 2102EP-C1-pLKO.1 or 2102EP-C1-shKDM6A + shKDM6B (sh6A + 6B) cells treated with PBS or 0.5 µM cisplatin for 6 h. In all cases, cells were harvested for RNA 24 h after cisplatin treatment. RNA was isolated with the RNeasy plus Mini Kit (Qiagen) and RNA sequencing was performed by the Roy J. Carver Biotechnology Center. RNA-Seq libraries were prepared using the TruSeq Stranded mRNA Sample Prep kit. The libraries were sequenced on a HiSeq 4000 using HiSeq 4000 sequencing kit version 1. Initial quality control was performed using FASTQC. Trimmomatic was used to remove low-quality bases from both ends LEADING ≤ 28 and TRAILING ≤ 28, respectively, with a minimum length of 30. The reads in FASTQ format were aligned to human genome assembly NCBI GRCh38.p14 using STAR aligner. Reads were counted and assigned to genes using featureCount. The “Limma” R package was used to identify differentially expressed genes [34]. Genes whose expression was not greater than 0.5 counts per million in at least 2 samples were removed and the resultant filtered expression matrix was TMM-normalized. Benjamini-Hochberg False Discovery Rate (FDR) was used to correct for multiple hypotheses. The “Enhanced Volcano” R package was used to visualize volcano plots. The RNA-seq datasets for the current study have been submitted to the NCBI Database of GEO Datasets under the accession numbers GSE278498.

### Downstream enrichment analysis

Gene Set Enrichment Analysis (GSEA) from the Broad Institute was performed to identify enriched gene sets [35]. GeneOverlap package from R Bioconductor was used to identify significant gene set overlap between common or exclusive gene expression changes between pLKO.1 control cells (pLKO.1 cisplatin-treated vs. pLKO.1 untreated), those genes basally regulated by KDM6A and KDM6B knockdown (sh6A + 6B untreated vs. pLKO.1 untreated), and those genes regulated by cisplatin in KDM6A and KDM6B knockdown cells (sh6A + 6B cisplatin-treated vs. sh6A + 6B untreated) and C2 gene sets from the MSigDB database [35, 36].

### Western analysis and real-time PCR

For Western analysis cells were lysed in radioimmune precipitation buffer and separated by SDS-PAGE. Antibodies to actin (MA1-744, Thermo Fisher), DNMT3B (HPA001595, Atlas Antibodies) Ubiquitin H2A-K119 (3240, Cell Signaling Technology), H3K27me3 (9733, Cell Signaling Technology), BMI1 (6964, Cell Signaling Technology), EZH2 (5246, Cell Signaling Technology) and histone H3 (ab1791, Abcam) were used. Total cellular RNA was isolated using the RNeasy Mini Kit (Qiagen), and complementary DNAs (cDNAs) were synthesized using High-capacity cDNA Synthesis Kit (Thermo Fisher Scientific). Quantitative real-time PCR assays were performed with PowerUp™ SYBR™ green master mix (Thermo Fisher Scientific) and the QuantStudio 3 Real-time System (Thermo Fisher Scientific). In all cases gene expression was normalized to β-actin. Primers for RT-PCR will be provided upon request.

### Statistics

Student’s t-tests and ANOVA were performed using GraphPad Prism 10. *p*-values indicative of non-significance (*p* > 0.05) and significance (*p* ≤ 0.05; *, *p* ≤ 0.01; **, *p* ≤ 0.001; *** and *p* ≤ 0.0001; ****) were determined. Mean and standard error of the mean were used to describe sample variability. For mouse tumor volume and weight measurements, a longitudinal analysis using a linear mixed model fit was performed with the R package TumGrowth [37]. Survival curves with generated in GraphPad Prism using a Kaplan-Meier log-rank (Mantel-Cox) test.

## Results

### Pharmacologic repression of the polycomb pathway by inhibition of polycomb methylase EZH2 with GSK-126 confers cisplatin resistance to TGCT cells but not other cancer cell types, while polycomb induction by inhibition of polycomb demethylases KDM6A and KDM6B with GSK-J4 sensitizes TGCT cells to cisplatin

**Fig. 1:**
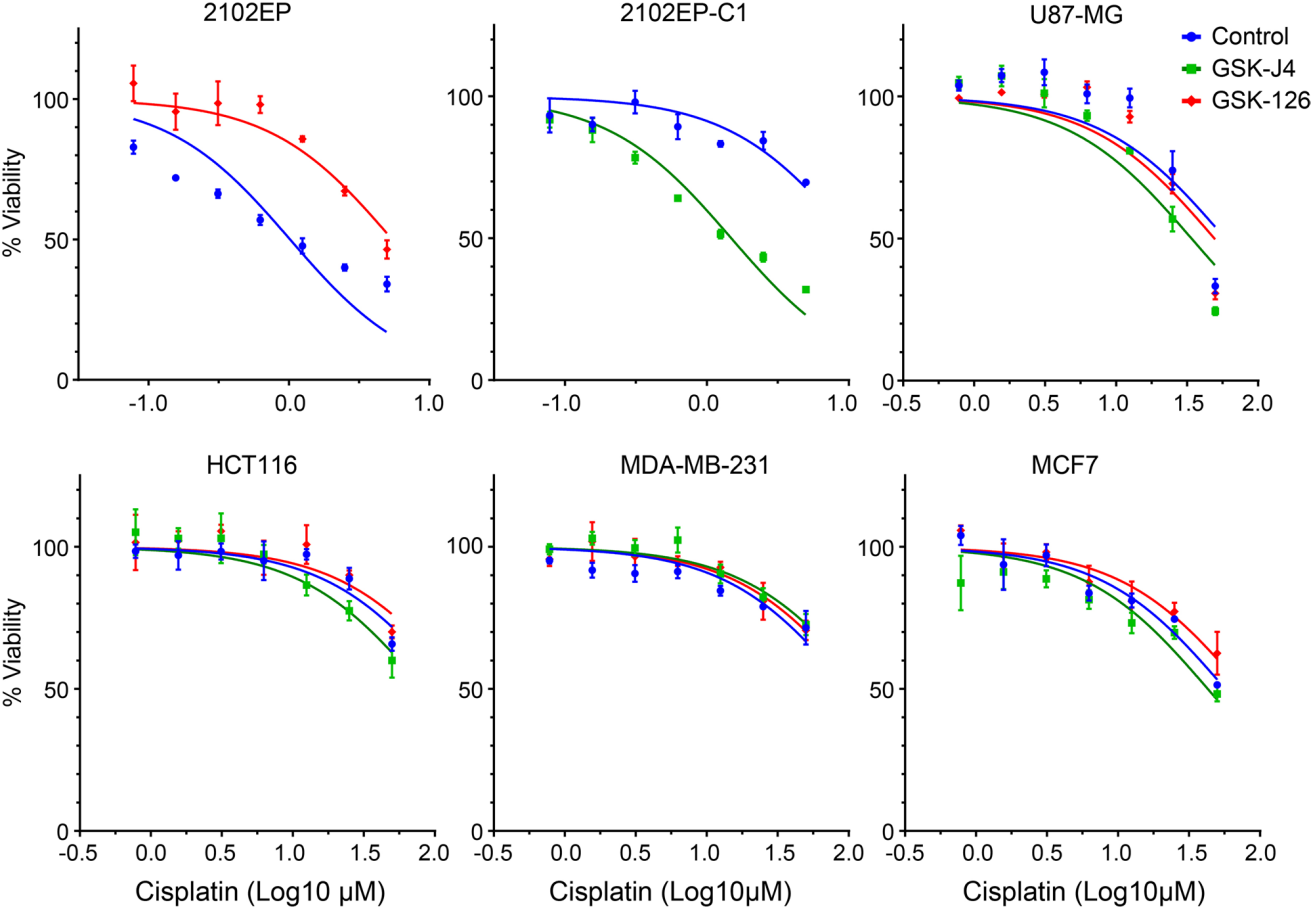
Pharmacologic inhibition or induction of the polycomb pathway alters cisplatin sensitivity in TGCT cells but not in glioblastoma, colon, and breast cancer cells. Parental TGCT cell line 2102EP was treated with EZH2 inhibitor GSK-126 (0.5 µM) for 3 days and cisplatin-resistant TGCT cell line 2102EP-C1 was treated with KDM6A/KDM6B inhibitor GSK-J4 (1.0 µM) for 3 days before 6-hour cisplatin treatments. Cells were assayed for viability 3 days later. U87-MG glioblastoma, HCT116 colon cancer, and MDA-MB-231 and MCF7 breast cancer cells were treated similarly, except cisplatin dosages were higher due to less inherent sensitivity to cisplatin compared to TGCT cells. See Supplementary Figure S1 for p values for Fig. 1

We showed previously that multiple isogeneic cisplatin-resistant TGCT cell lines had a reduction in the polycomb repressive mark H3K27me3 and reduced levels of polycomb repressive complex 2 (PRC2) component EZH2 and polycomb repressive 1 (PRC1) component BMI1 with a corresponding induction of polycomb target genes [32]. We also show previously in multiple cell lines and in Fig. 1, that inhibition of polycomb signaling with the EZH2 inhibitor GSK-126 confers cisplatin resistance in parental 2102EP TGCT cells, while potentiation of polycomb signaling with an inhibitor of the H3K27me3 demethylases KDM6A and KDM6B, called GSK-J4, confers cisplatin sensitization in cisplatin-resistant 2102EP-C1 TGCT cells [32]. Note, cells were pretreated with GSK-J4 and GSK-126 for 3 days at doses previously established to not affect cell proliferation or viability as single agents [32]. To address whether polycomb has the ability to generally alter the cisplatin sensitivity of cancer cells, we tested the effects of GSK-126 and GSK-J4 on a number of non-TGCT cancer cell lines, including breast cancer cells MCF-7 and MDA-231, colon cancer cells HCT116, and glioblastoma cells U87-MG. GSK-126 and GSK-J4 had minimal effects on cisplatin sensitivity of these cell lines, suggesting that cisplatin sensitivity of TGCT cells may be uniquely altered by polycomb (Fig. 1). The p values for Fig. 1 are provided in Supplementary Figure S1.

### Knockdown of EZH2 and BMI1 confers cisplatin resistance in wild-type TGCT cells, while knockdown of KDM6A/KDM6B sensitizes TGCT cells to cisplatin *in vitro*

**Fig. 2:**
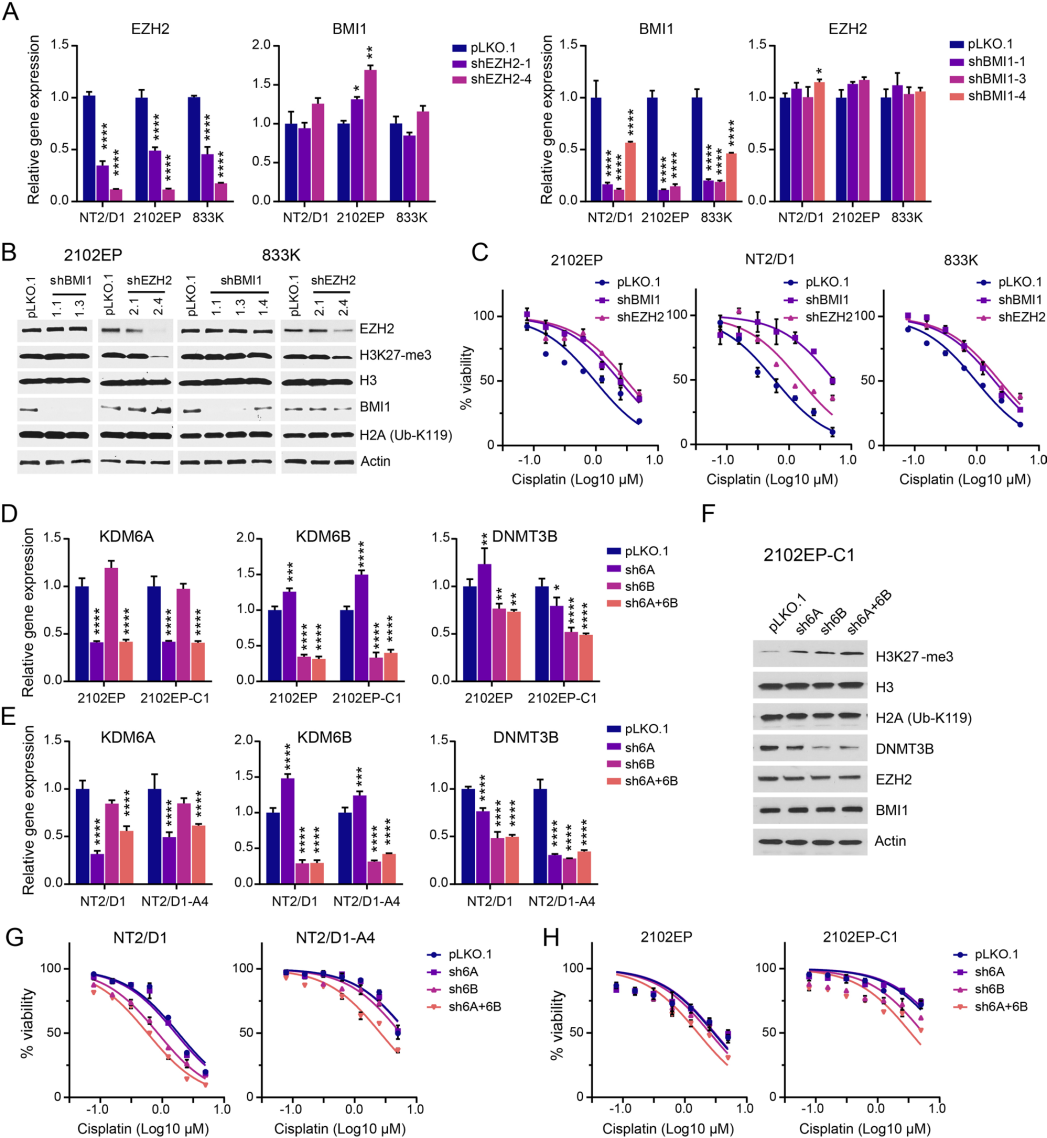
Knockdown of polycomb components EZH2 and BMI1 confer cisplatin resistance in TGCT cells, while knockdown of polycomb demethylases KDM6A/KDM6B sensitizes TGCT cells to cisplatin. (**A**) RT-PCR demonstrating efficient knockdown of EZH2 and BMI1 in TGCT cells NT2/D1, 2102EP, and 833 K. (**B**) Western blot demonstrating efficient knockdown of EZH2 and BMI1 in 2012EP and 833 K cells and repression of H3K27me3 levels with EZH2 knockdown. (**C**) Cell proliferation and viability assays of control pLKO.1 and EZH2- and BMI1-knockdown cells treated with cisplatin. (**D**, **E**) RT-PCR demonstrating single and dual KDM6A- and KDM6B-knockdown in 2102EP, 2012EP-C1, NT2/D1, and NT2/D1-A4 cells and repression of DNMT3B expression upon KDM6A/KDM6B-knockdown. (**F**) Western blot demonstrating induction of H3K27me3 and repression of DNMT3B expression with single and dual knockdown of KDM6A and KDM6B in 2102EP-C1 cells. (**G**, **H**) Cell proliferation and viability assays of control pLKO.1 and KDM6A/KDM6B-knockdown cells treated with cisplatin. See Supplementary Figure S1 for p values for Fig. 2C, G and H

We next tested whether genetic perturbation of the polycomb pathway could alter the cisplatin sensitivity of TGCT cells. Inhibition of polycomb signaling by EZH2- or BMI1-knockdown conferred cisplatin resistance in parental cisplatin-sensitive TGCT 833 K, 2102EP, and NT2/D1 cells (Fig. 2A-C). Note, while BMI1 shRNA decreased BMI1 levels, it did not alter biological target Ub-H2AK119, while EZH2-knockdown did repress H3K27me3 levels as expected (Fig. 2B). This implies that BMI1 may have a Ub-H2AK119-independent effect on TGCT cells. Reciprocally, induction of polycomb signaling with dual knockdown of KDM6A and KDM6B resulted in cisplatin sensitization in both cisplatin-sensitive NT/2D1 and 2102EP cells and cisplatin-resistant counterparts, NT2/D1-A4 and 2102EP-C1 cells (Fig. 2D-H).

In prior work, we found an interconnected relationship between alterations in DNA methylation mediated by DNMT3B and H3K27me3-mediated polycomb signaling [31, 33]. For example, DNMT3B is overexpressed in cisplatin resistant TGCT cells, while H3K27me3 levels are decreased compared to parental cells [33]. Also, DNMT3B-knockdown sensitized parental TGCT cells to cisplatin and induced BMI1, EZH2, and H3K27me3 levels [33]. Consistent with this interconnected relationship KDM6A/KDM6B-knockdown repressed expression of DNMT3B in TGCT cells (Fig. 2D-F). The p values for Fig. 2C, G and H are provided in Supplementary Figure S1.

### H3K27me3 specific histone demethylase inhibitor GSK-J4 and KDM6A and KDM6B dual knockdown dramatically synergizes with cisplatin to promote TGCT inhibition and regression *in vivo*

**Fig. 3:**
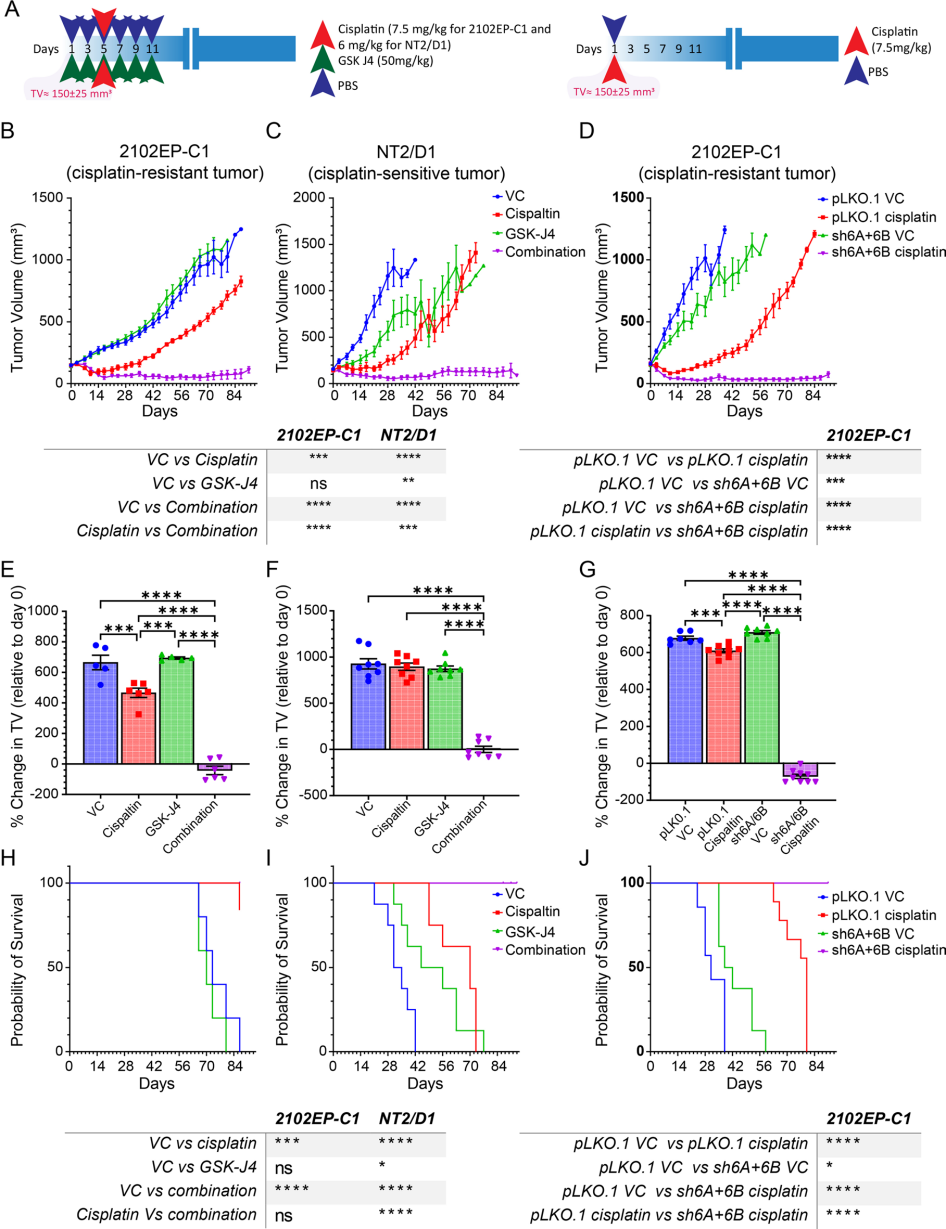
KDM6A/KDM6B inhibitor GSK-J4 and dual KDM6A/KDM6B-knockdown sensitize human TGCT tumor xenografts to cisplatin. (**A**) Schematic of GSK-J4, cisplatin, or GSK-J4 + cisplatin treatment schedule for cisplatin-resistant 2102EP-C1 and cisplatin-sensitive NT2/D1 cells xenografts (left) and schematic of cisplatin treatment schedule for KDM6A/KDM6B dual knockdown or control 2102EP-C1 cells (right). (**B**-**J**) Depicted are tumor volume, percent change in tumor volume from the day prior to treatment initiation (day 0), and survival of mouse xenografts for 2102EP-C1 and NT2/D1 tumors treated with GSK-J4, cisplatin, or GSK-J4 + cisplatin (left) or xenograft tumors of KDM6A/KDM6B dual knockdown or control 2102EP-C1 cells (right). TV, tumor volume

To assess whether GSK-J4 could potentiate cisplatin sensitivity to TGCT cells in vivo, we performed xenograft studies with cisplatin-resistant 2102EP-C1 and cisplatin-sensitive NT2/D1 cells. While GSK-J4 alone had minimal effects on TGCT growth, GSK-J4 produced a dramatic synergistic interaction with cisplatin treatment with evidence of tumor regression (Fig. 3). Mice treated with a single round of GSK-J4 and cisplatin therapy remained tumor-free at the end of the experiment (90 days). Note that the dose of cisplatin was decreased in cisplatin-sensitive NT2/D1 cells in order to observe a potentiation effect with GSK-J4 (Fig. 3). This suggests that GSK-J4 may not only be able to restore cisplatin sensitivity to resistant cells but may also be a strategy for cisplatin-sparing therapy for cisplatin-sensitive tumors. GSK-J4 or GSK-J4 plus cisplatin had minimal toxicity as assessed by total body weight (Supplemental Figure S1). In contrast, while not as dramatic, EZH2 inhibitor GSK-126 conferred cisplatin resistance in TGCT xenografts (Supplemental Figure S2).

To address whether GSK-J4 may have off-target effects, we performed further xenografts with cisplatin-resistant 2102EP-C1 cells after dual knockdown of the intended targets of GSK-J4, KDM6A and KMD6B, and compared the results to control cells. Similar to GSK-J4, KDM6A/ KDM6B-knockdown minimally effected basal tumor growth but dramatically potentiated the effects of cisplatin, again with evidence of tumor regression, and all mice remained tumor-free for over 90 days after a single dose of cisplatin (Fig. 3). Together these results suggest that pharmacologic and genetic activation of the polycomb pathway in TGCTs has strong cisplatin potentiated effects in vivo with low overall toxicity. The dramatic in vivo effects, as compared to in vitro effects of GSK-J4 and KDM6A/KDM6B-knockdown, suggest that host and/or tumor microenvironment contributions may be occurring during cisplatin sensitization.

### Transcriptome analysis of GSK-J4 and KDM6A/KDM6B-knockdown cells reveals the importance of basal activation of polycomb and p53 signaling in cisplatin sensitization

To investigate potential mechanisms responsible for the cisplatin sensitization effects of polycomb demethylase targeting in TGCT cells, we performed RNA-seq analysis of cisplatin-resistant NT2/D2-A4 and 2102EP-C1 cells untreated or treated with cisplatin and GSK-J4 alone or in combination and also untreated or cisplatin treated control 2102EP-C1-PLKO.1 and isogeneic dual 2102EP-C1-shKDM6A + 6B-knockdown cells. Note, in cell viability/cytotoxic assays, cells were treated with cisplatin for 6 h and assayed 3 days later to mimic clinical usage (peak cisplatin plasma concentration over a short amount of time) [1, 2]. As we have documented before, for transcriptomic analysis, the post-cisplatin time point was shortened to 24 h to better assess proximal alterations in gene expression not associated with active cell death [25, 38].

**Fig. 4:**
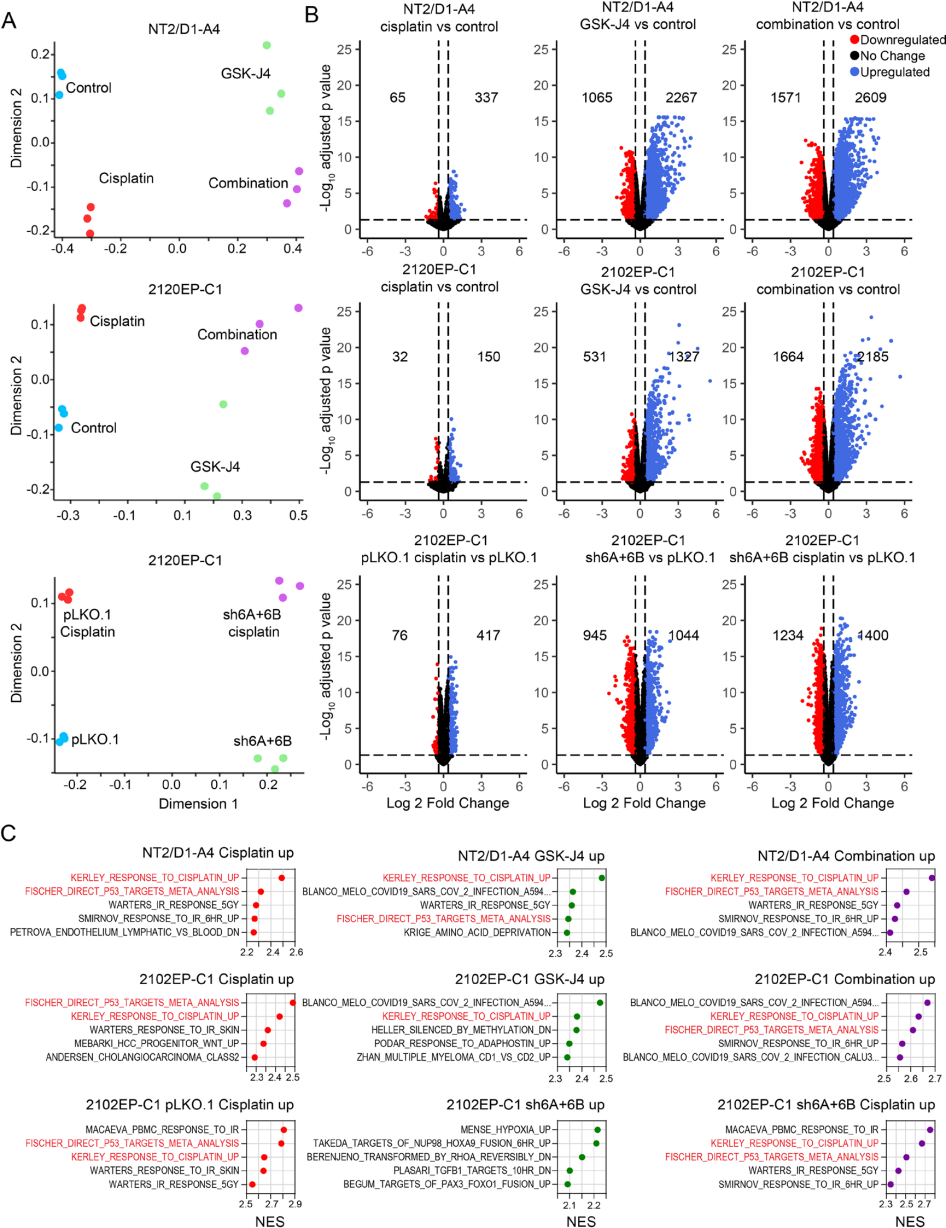
Transcriptome analysis of GSK-J4 treated and KDM6A/KDM6B knockdown cells reveals the importance of basal activation of gene expression and p53 signaling in cisplatin sensitization. (**A**) Multidimensional scaling (MDS) plots of the three RNA-seq experiments NT2/D1-A4 and 2102EP-C1 cells treated with GSK-J4, cisplatin, or GSK-J4 + cisplatin and KDM6A/KDM6B dual knockdown or control 2102EP-C1 cells treated with cisplatin. (**B**) Enhanced volcano plots of the three RNA-seq experiments. For the first two experiments, each treatment (cisplatin, GSK-J4, or the combination) is compared to untreated vehicle control. For the third experiment, PLK control + cisplatin, untreated sh6A + 6B, and sh6A + 6B + cisplatin cells are compared to untreated PLK cells. Significant cutoff is fold-change > 1.3 and FDR < 0.05. The number of up- and downregulated genes are provided. (**C**) Gene set enrichment analysis (GSEA) results corresponding to the volcano plot comparisons for upregulated genes. Top 5 gene sets as determined by normalized enrichment score (NES) from the MSigDB C2 collection are provided. The top 20 gene sets enriched for upregulated and downregulated genes for each experimental arm for all three RNA-seq experiments are provided in Supplemental Table S2. Red text highlights p53 target gene sets, including KERLEY_RESPONSE_TO_CISPLATIN, which we previously identified to be a p53 dominate gene set in cisplatin treated NT2/D1 cells [38]

**Fig. 5:**
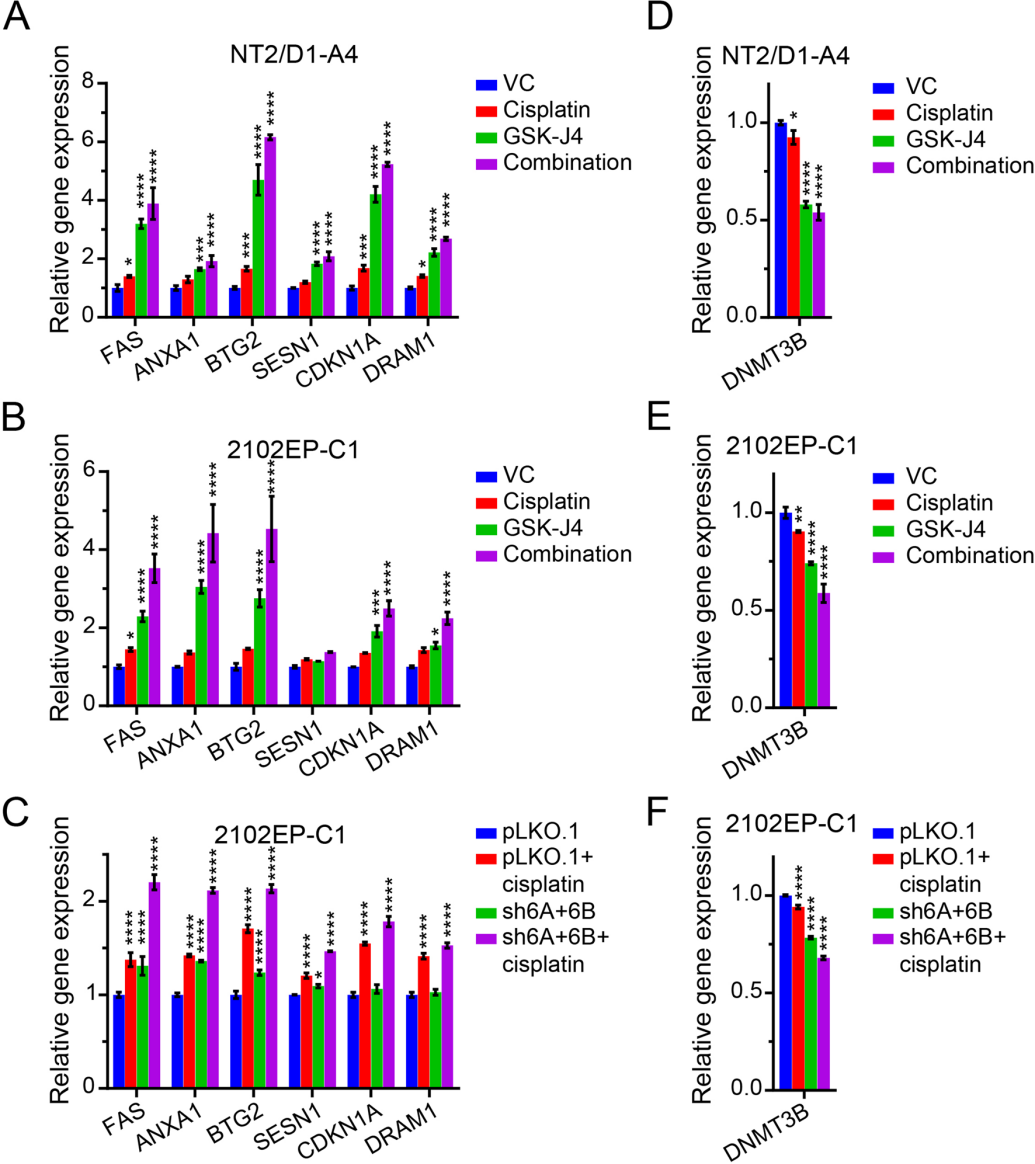
Polycomb demethylase targeting potentiates p53 target gene activation and represses DNMT3B expression in TGCT cells. (**A**-**C**) Expression of select p53 target genes across the 4 experimental arms of the three RNA-seq experiments of Fig. 4. (**D**-**F**) DNMT3B expression across the 4 experimental arms of the three RNA-seq experiments of Fig. 4

Multidimensional scaling (MDS) plots demonstrated a clear separation of the experimental groups and a tight grouping of biological replicates within groups (Fig. 4A). Volcano plots and GSEA revealed that cisplatin treatment of cisplatin-resistant NT2/D1-A4, 2102EP-C1, and 2102EP-C1-PLKO.1 control cells had a restricted pattern of gene alterations dominated by upregulated p53 target genes (Fig. 4B-C), as we have noted previously in transcriptome analysis of TGCT cells treated with cisplatin [25, 38]. Of note, the degree of transcriptional changes was substantially reduced in comparison to parental cisplatin-sensitive cells (data not shown). In contrast, GSK-J4-pretreated NT2/D1-A4 and 2102EP-C1 cells and untreated 2102EP-C1-shKDM6A + 6B cells had a substantially more robust transcriptional response to cisplatin, again dominated by upregulated p53 target genes (Fig. 4B-C). Interestingly, GSK-J4 treatments alone and KDM6A/KDM6B-knockdown alone in 2102EP-C1-shKDM6A + 6B cells demonstrated substantial alterations in gene expression. Upregulated genes for GSK-J4 alone treatments were again dominated by p53 target genes (Fig. 4C and Fig. 5A-B) despite the fact that the GSK-J4 treatments were not toxic or growth-inhibitory in long-term assays (32 and data not shown). This basal p53 target gene effect was less prominent in 2102EP-C1-shKDM6A + 6B cells but was still evident in a narrower subset of p53 target genes (Fig. 5C). In contrast, downregulated genes had a more diverse gene set enrichment pattern among the experiments, including gene sets involving mRNA splicing, DNA methylation, and histones (Supplemental Table S2). The top 20 gene sets enriched for upregulated and downregulated genes for each experimental arm for all three RNA-seq experiments are provided in Supplemental Table S2.

High levels of OCT4 have been shown to correlate with cisplatin sensitivity in TGCT cells mediated in part through induction of pro-apoptotic factor NOXA [39]. Also, KDM6A has been shown to interact directly with OCT4 to promote OCT4 target gene expression during chemotherapy in breast cancer cells [40]. This prompted us to investigate whether targeting of KDM6A/6B in TGCT cells alters Oct4 target gene expression. Pharmacologic and genetic inhibition of KDM6A/6B had decreased expression of several OCT4 target genes (Supplemental Figure S3) consistent with KDM6A being a cofactor of OCT4. However, repression of OCT4 signaling would be predicted to cause resistance to cisplatin in TGCT cells not sensitization [39]. Interestingly, despite being reported as being induced by OCT4 in TGCT cells, NOXA was upregulated in TGCT cells upon inhibition of KDM6A/6B (Supplemental Figure S3). Again, consistent with an interconnected relationship between alterations in DNA methylation mediated by DNMT3B and H3K27me3-mediated polycomb signaling [31, 33], pharmacologic inhibition of KDM6A/KDM6B with GSK-J4 or KDM6A/KDM6B knockdown repressed expression of DNMT3B in both cisplatin-sensitive and -resistant TGCT cells (Fig. 5D-F).

### Transcriptome analysis of KDM6A/KDM6B-knockdown cells reveals cisplatin sensitization is associated with alterations in chromatin remodeling genes upon cisplatin treatment

**Fig. 6:**
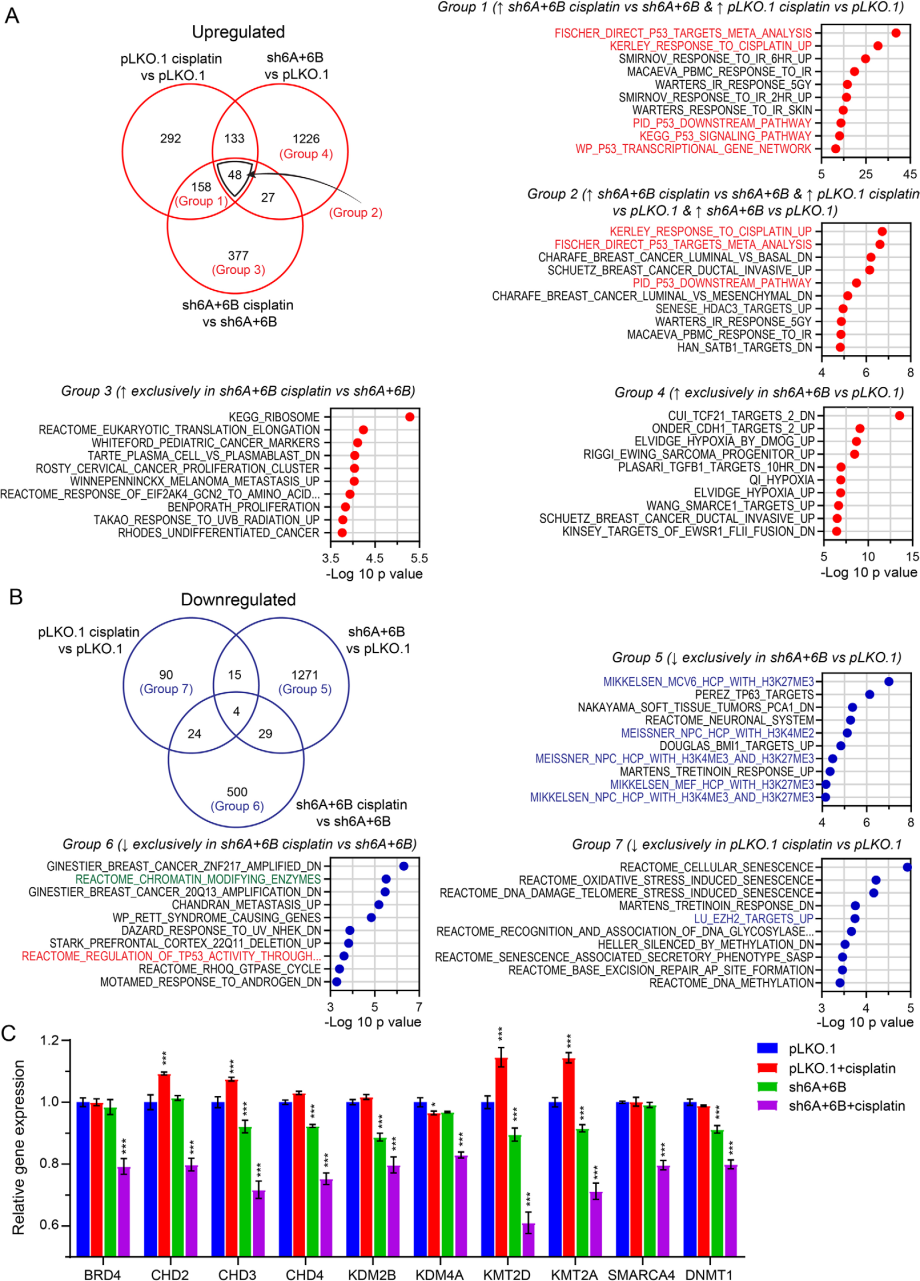
Transcriptome analysis of KDM6A/KDM6B-knockdown cells reveals cisplatin sensitization is associated with alterations in chromatin remodeling genes upon cisplatin treatment. (**A**) Venn diagram comprised of comparing genes upregulated in PLKO.1 cisplatin-treated vs. PLKO.1 untreated, sh6A/KDM6B untreated vs. PLKO.1 untreated, and sh6A/KDM6B cisplatin vs. sh6A/6B untreated. Also depicted are gene set enrichment analysis (GSEA) results corresponding to indicated comparison groups. Top 10 gene sets from GeneOverlap analysis as determined by p value from the MSigDB C2 collection are provided. P53 target gene collections are highlighted. malignant peripheral nerve sheath tumors (MPNSTs), **B**) Venn diagram comprised of comparing genes downregulated in PLKO.1 cisplatin-treated vs. PLKO.1 untreated, sh6A/6B untreated vs. PLKO.1 untreated, and sh6A/6B cisplatin-treated vs. sh6A/6B untreated. Also depicted are GeneOveralp results corresponding to indicated comparison groups. Top 10 gene sets as determined by p value from the MSigDB C2 collection are provided. The REACTOME_CHROMATIN_MODIFYING_ENZYMES gene set is highlighted. (**C**) Expression of select chromatin-modifying enzymes and proteins across the 4 experimental arms of the RNA-seq experiment. P53 target gene sets are in red text, H3K27me3/polycomb gene sets are in blue text, REACTOME_CHROMATIN_MODIFYING_ENZYMES gene set is in green text

To gain a broader insight into the role of polycomb demethylase targeting in cisplatin sensitization of TGCT cells, we further analyzed the KDM6A/KDM6B-knockdown plus cisplatin treatment in 2102EP-C1 cells experiment by identifying subsets of unique and overlapping upregulated and downregulated genes from three comparisons, those genes regulated by cisplatin in PLKO.1 control cells (PLKO.1 cisplatin-treated vs. PLKO.1 untreated), those genes basally regulated by KDM6A/KDM6B-knockdown (shKDM6A + 6B untreated vs. PLKO.1 untreated), and those genes regulated by cisplatin in KDM6A/KDM6B-knockdown cells (shKDM6A + 6B cisplatin-treated vs. shKDM6A + 6B untreated) (Fig. 6A-B). Venn diagrams were generated with a cutoff of > 1.2 fold-change with FDR < 0.001 and GeneOverlap analysis with Fisher exact tests were performed against the 5529 curated sets from the Broad MSigDB C2 collection (Fig. 6A-B). Gene lists from Venn diagram analysis and GeneOverlap results are provided in Supplemental Table S3 and Supplemental Table S4. Upregulated genes were again dominated by p53 target genes with p53 gene sets enriched for genes commonly upregulated in both PLKO.1 and 2102EP-C1-shKDM6A + 6B cells treated with cisplatin (Group 1), and genes upregulated by cisplatin in both cells but also basally upregulated upon KDM6A/KDM6B-knockdown (Group 2) (Fig. 6A). This analysis again suggests that targeting polycomb demethylases basally modifies the p53 pathway in TGCT cells to sensitize these cells to cisplatin. Gene sets enriched exclusively for genes upregulated in shKDM6A + 6B cells treated with cisplatin include several related to cancer (Group 3) (Fig. 6A). Finally, gene sets enriched exclusively for upregulated genes in untreated KDM6A/KDM6B knockdown cells compared to untreated PLKO.1 were involved in hypoxia and E-cadherin (CDH1) signaling (Group 4).

In contrast, there was strong enrichment for gene sets involved in H3K27me3 and polycomb signaling for gene exclusively downregulated in KDM6A/KDM6B-knockdown cells, as would be expected by knocking down polycomb demethylases (Group 5) (Fig. 6B). Interestingly, we found a gene set of chromatin-modifying enzymes and proteins [41] that were only downregulated in 2102EP-C1-shKDM6A + 6B cells treated with cisplatin (Group 6) (Fig. 6B-C). This included bromodomain protein BRD4, ATP-dependent chromatic remodeler SMARCA4, and cassettes of lysine demethylases (KDMs), chromodomain helicase DNA binding proteins (CHDs), and lysine methyltransferases (KMTs) (Fig. 6C) [41]. This suggests that targeting KDM6A/KDM6B sets the stage for further cisplatin-mediated chromatin remodeling in TGCT cells. Gene sets enriched in genes exclusively downregulated by cisplatin in cisplatin-resistant PLKO.1 cells were related to senescence (Group 7).

## Discussion

Due largely to a dearth of driver mutations in contrast to many solid tumors, there have been no effective targeted therapies developed for TGCTs, which are mainly treated with cisplatin-based chemotherapies developed over 4 decades ago [10, 11]. While cisplatin has transformed metastatic testicular cancer from a deadly, to in the majority of cases, a curable disease, there are no effective backup therapies for the 15% of cisplatin-refractory/resistant patients who typically die from progressive disease [1, 2]. Further, curative cisplatin therapy results in acute and life-long toxicities, which are especially pertinent to the adolescent and young adult TGCT patient population [4, 5]. Strategies to decrease cisplatin exposure would likely lead to improved quality of life for these patients. In contrast to genetic alterations, recent evidence suggests that epigenetics is a major driving factor for TGCT formation, progression, and response or resistance to chemotherapy [7, 8, 42]. Hence, targeting epigenetic pathways with “epidrugs” is one potential relatively unexplored strategy to advance TGCT treatment beyond cisplatin.

In this report, we preclinically validate targeting polycomb demethylases KDM6A and KDM6B with epidrug GSK-J4 for the treatment of both cisplatin-sensitive and -resistant TGCTs. While GSK-J4 had minimal effects alone on TGCT tumor growth in vivo, it dramatically sensitized cisplatin-sensitive and -resistant TGCTs to cisplatin. We validated KDM6A/KDM6B as the target of GSK-J4 since KDM6A/KDM6B genetic depletion had a remarkably similar effect to GSK-J4 on cisplatin-mediated anti-tumor activity and transcriptome alterations. Pharmacologic and genetic targeting of KDM6A/KDM6B potentiated or primed the p53-dominant transcriptional response to cisplatin, with also evidence for basal activation of p53. Further, several chromatin modifier gene families were repressed with cisplatin only in KDM6A/KDM6B-targeted cells, implying that KDM6A/KDM6B inhibition sets the stage for extensive chromatin remodeling of TGCT cells upon cisplatin treatment. Another interesting finding of our study was the contrast between the dramatic cisplatin sensitization effect of GSK-J4 in vivo compared to cell culture. This suggests that perhaps there is priming of anti-tumor microenvironment and innate host immune mechanisms with GSK-J4 against TGCTs, a premise that is worthy of future study.

Several lines of evidence suggest that TGCTs may be particularly driven by epigenetic alterations [6–9]. TGCT are thought to derive from aberrantly differentiated primordial germ cells during a stage in development where the male germ line undergoes a dynamic wave of DNA methylation erasure. Issues that impact the microenvironment of male germ cell development *in utero*, including cryptorchidism, hypospadias, impaired spermatogenesis, high estrogen exposure, and exposure to endocrine disrupting chemicals, have been associated with TGCTs [43–47]. Germline genetic disorders of sex development associated with fetal androgen insufficiency are also associated with an increased risk of germ cell malignancy [48]. Further, TGCTs have a very low mutational burden and a low frequency of driver oncogenic or tumor-suppressive mutations, especially in nonseminomas [10, 11]. Evidence from our lab and others has shown that TGCT cells hyperactive p53 during cisplatin responses [38, 49–51]. The current work also suggests an important role for p53 in cisplatin sensitization of resistance cells upon KDM6A/KDM6B-targeting as p53 target gene expression was potentiated and basally activated. Interestingly, other cancer types we tested, including MCF7 cells with wild-type p53, did not show a high degree of cisplatin sensitization with GSK-J4. In future preclinical work it will be to important perform detailed characterization of the epigenetic status of p53 target genes upon KDM6A/KDM6B inhibition and to assess whether cisplatin sensitive cells share these same features as we have evidence in Fig. 3 that KDM6A/6B targeting also sensitizes parental TGCT cells to cisplatin. This may be related the unique epigenetics of TGCTs due to their developmental origins and that TGCTs appear uniquely driven by epigenetics compared to other cancers. Hence TGCTs may be uniquely vulnerable to epidrugs including those that target KDM6A/KDM6B. Although we have focused on post target mechanisms of cisplatin sensitization, KDM6A/KDM6B inhibition could potentially be mediating pre and on target effects of cisplatin in TGCT cells including altering cisplatin metabolism or altering the accessibility of cisplatin to DNA to alter DNA adduct formation.

Due to their developmental origins, TGCTs may have unique and more open, embryonic stem cell-like chromatin as compared to somatic cell-derived solid tumors, which may make TGCTs uniquely vulnerable to certain epidrugs [9, 52]. We and others have shown that TGCT cells are exquisitely sensitive to hypomethylating agents (HMAs) at very low doses that are dependent on intrinsically high levels of DNMT3B [24–28]. Further, pretreatment with HMAs can restore cisplatin sensitivity to cisplatin-resistant TGCT cells [24–26]. Two recent clinical trials suggest that HMAs may have clinical activity in the setting of cisplatin refractory TGCTs [29, 30]. Utilizing isogenic HMA- and cisplatin-resistant cell lines, we found that sensitivity/resistance to HMAs and cisplatin appear to be mechanistically linked by epigenetic remodeling involving DNA methylation and the polycomb pathway. Namely, there is a common set of polycomb target genes upregulated in cisplatin-resistant TGCT cells due to a shift in DNA methylation linked to high levels of DNMT3B [31–33]. Further, DNMT3B genetic targeting induced H3K27me3, EZH2, and BMI1 and resulted in increased sensitivity to cisplatin [33]. The data presented here provides further evidence for this linkage as targeting KDM6A/KDM6B was associated with a decrease in DNMT3B levels along with increasing cisplatin sensitivity. Hence, HMAs and GSK-J4 may be essentially targeting the same pathway vulnerability in TGCTs, with GSK-J4 having perhaps the theoretical advantage of being less genotoxic compared to HMAs, which incorporate into DNA and form protein adducts with DNMTs. The precise mechanism for how DNMT3B and polycomb are linked to regulate cisplatin sensitivity of TGCTs will require further study.

This report suggests that GSK-J4 may have cisplatin-sensitization properties for cisplatin-sensitive as well as cisplatin-resistant TGCT patients. The role of polycomb in cancer is complex [19]. In solid tumors, polycomb has mainly been associated with oncogenesis and poor outcomes, which has spurred the clinical development of EZH2 inhibitors [53]. However, loss-of-function PRC2 mutations also occur in a subset of tumor types, including malignant peripheral nerve sheath tumors (MPNSTs), pediatric gliomas, and T-cell acute lymphoblastic leukemia [17–19]. This highlights the complex role of polycomb in tumorigenesis. This complexity extends to whether polycomb-mediated epigenetic changes are associated with cancer drug resistance [20–23]. Targeting polycomb demethylases has not been well developed clinically for cancer therapy compared to EZH2 targeting, with a limited number of preclinical reports of GSK-J4 having anti-tumor activity [15, 54]. To our knowledge, GSK-J4 has not entered the clinic. Whether GSK-J4 or other KDM6A/KDM6B inhibitors have acceptable toxicity profiles will be important to ascertain, as we have only assessed toxicity by body weight. Also, cisplatin is typically given in 3–4 cycles, whether multiple treatments of KDM6A/KDM6B inhibitors lead to the development of resistance is unknown. It is noteworthy that in our studies GSK-J4 and KDM6A/KDM6B-targeting had minimal effects alone, suggesting that a therapeutic window may exist for combination therapy in those tumors like TGCTs that already have a heightened sensitivity to cisplatin. However, TGCTs appear to have unique epigenetics due to their developmental origins and TGCTs appear uniquely driven by epigenetics compared to other cancers.

## Conclusions

The biology of testicular germ cell tumors appears to be especially driven by epigenetic mechanisms suggesting that they may be highly sensitive to epidrugs. Our findings preclinically validate targeting polycomb demethylases KDM6A/KDM6B as a potent pharmacologic strategy for treating cisplatin-resistant TGCTs that warrants further preclinical and clinical investigation.

## Electronic supplementary material

Below is the link to the electronic supplementary material.

Supplementary Material 1

Supplementary Material 2

Supplementary Material 3

Supplementary Material 4

Supplementary Material 5

## Data Availability

The RNA-seq datasets generated and/or analyzed during the current study are available in the NCBI Database of GEO Datasets under the accession number GSE278498. All other data generated or analyzed during this study are available from the corresponding authors on reasonable request.

## Abbreviations

CHDs: chromodomain helicase DNA binding proteins
GSEA: gene set enrichment analysis
HMAs: DNA hypomethylating agents
KDMs: lysine demethylases
KMTs: lysine methyltransferases
MPNSTs: malignant peripheral nerve sheath tumors
PRCs: polycomb repressive complexes
PRC1: polycomb repressive 1
PRC2: polycomb repressive complex 2
TGCTs: testicular germ cell tumors
