# Supplementary figures and images for: Refractory testicular germ cell tumors are highly sensitive to the targeting of polycomb pathway demethylases KDM6A and KDM6B
Source: Cell Commun Signal. 2024 Oct 31;22:528. doi: 10.1186/s12964-024-01912-3 (PMC11529429; doi:10.1186/s12964-024-01912-3)

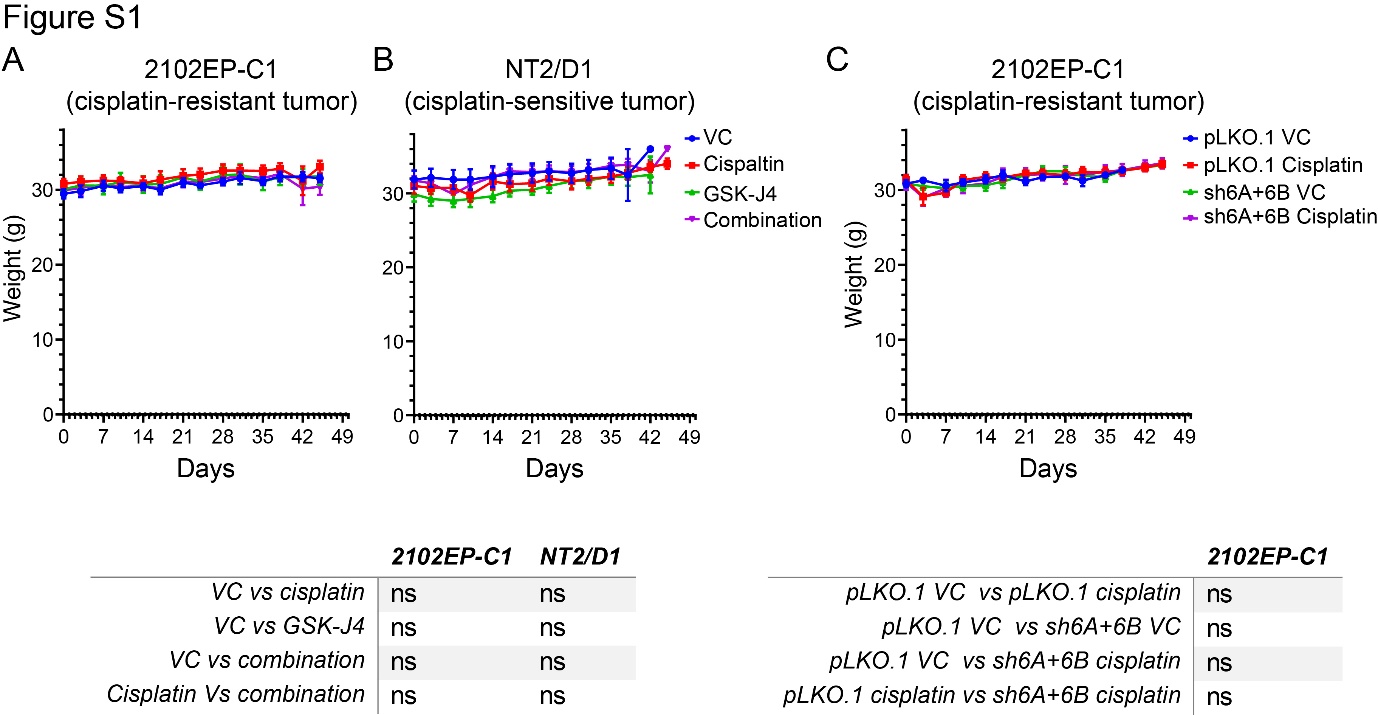


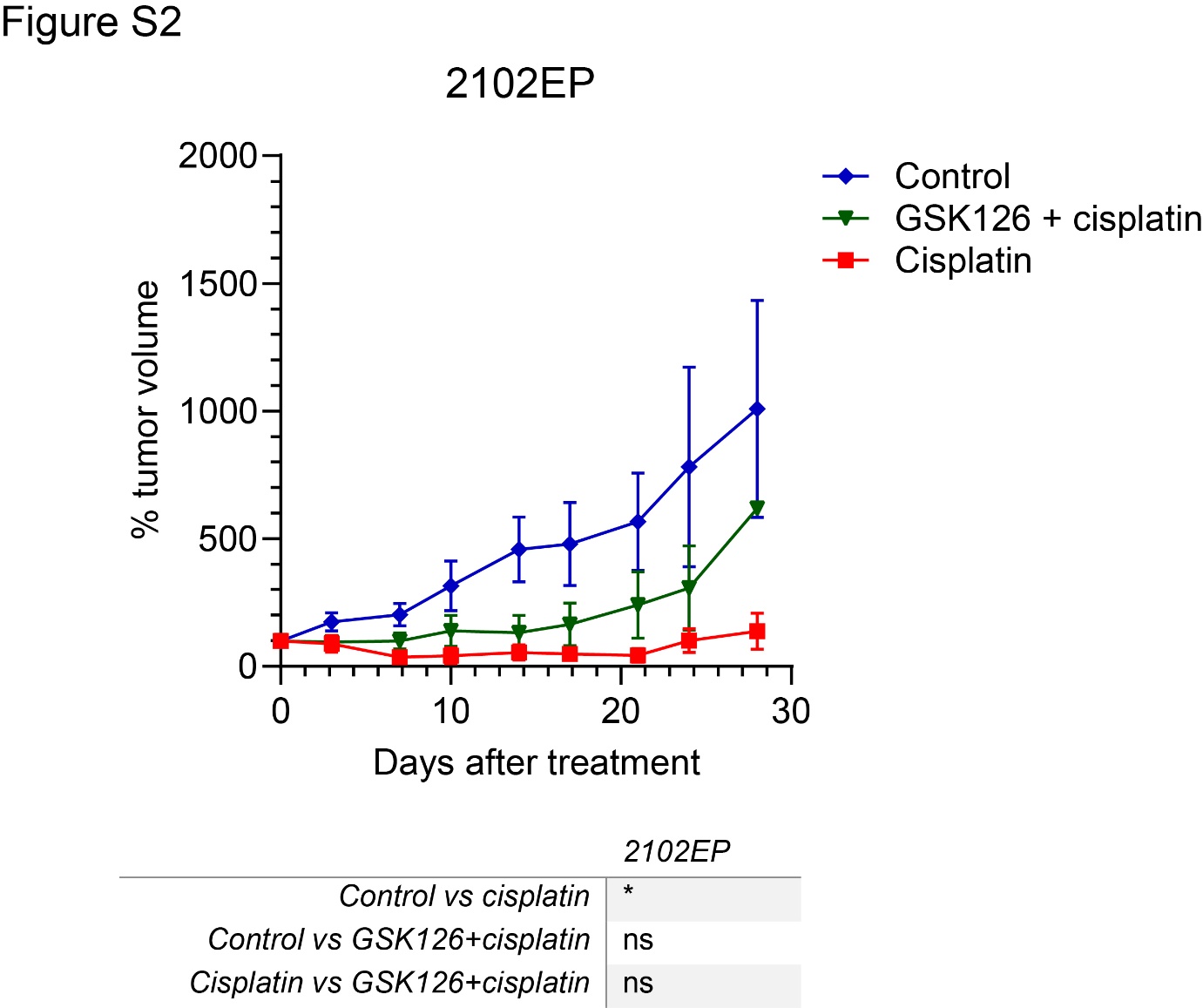


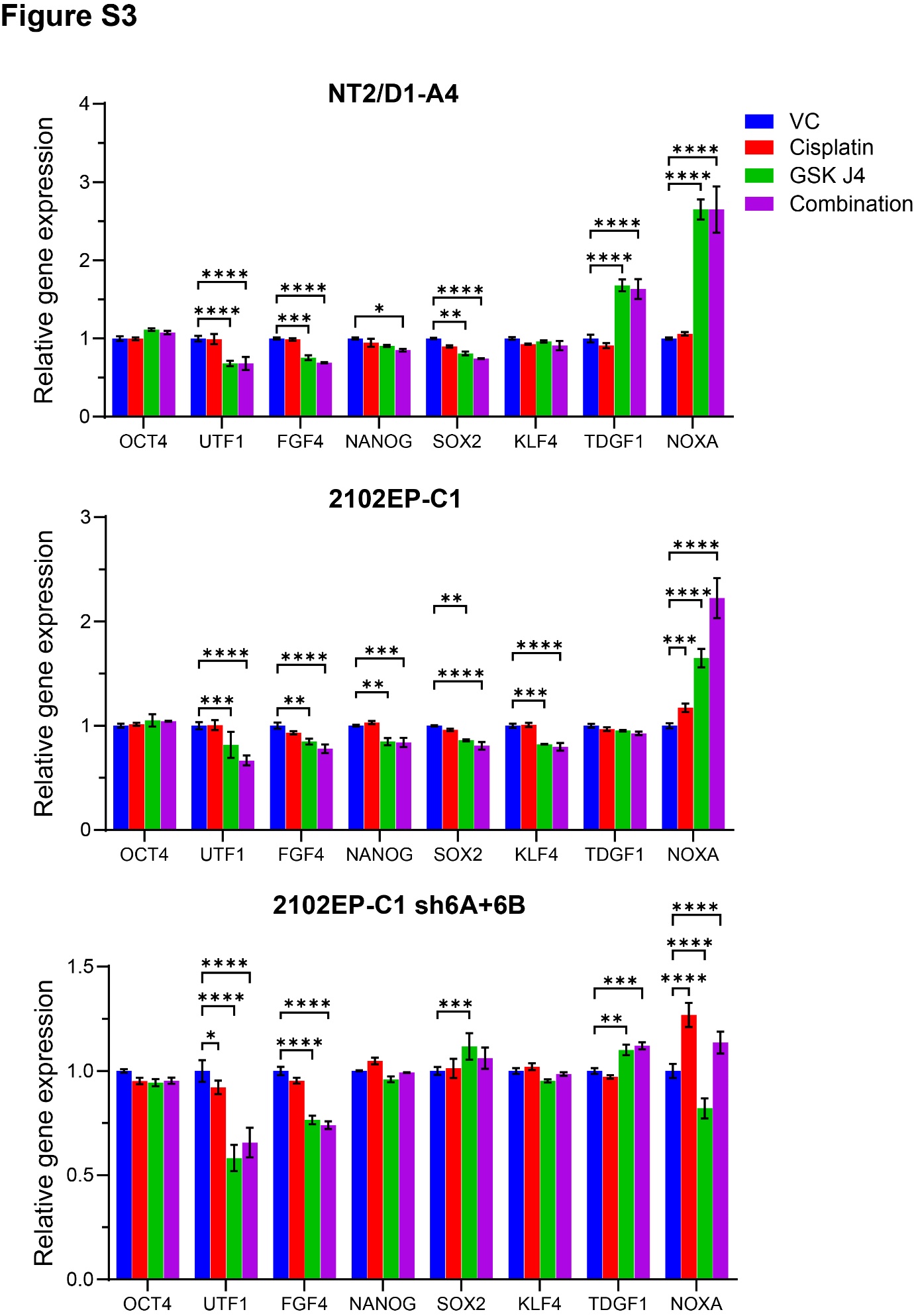

Supplement: Supplementary file 5 — Supplementary Material 5 [file 12964_2024_1912_MOESM5_ESM.docx]
